# Ectopic Expression of the *ydaS* and *ydaT* Genes of the Cryptic Prophage Rac of *Escherichia coli* K-12 May Be Toxic but Do They Really Encode Toxins?: a Case for Using Genetic Context To Understand Function

**DOI:** 10.1128/mSphere.00163-18

**Published:** 2018-04-25

**Authors:** Michael G. Jobling

**Affiliations:** a University of Colorado Anschutz Medical Campus, Aurora, Colorado, USA; University of Iowa

**Keywords:** prophage, regulatory cascades

## LETTER

It was welcoming to read in *mSphere* the two recent publications (1, 2) from collaborative groups on their work on the essentiality and biology of the Escherichia coli Rac prophage putative repressor, RacR. My interest in the *rac* locus and its regulation stems from my recent demonstration that both the heat-labile and pertussis toxin-like enterotoxins of type II enterotoxigenic E. coli are encoded within full-sized Rac-like prophages (3). Over a decade ago, a computer algorithm (4) designed to identify potential toxin-antitoxin gene pairs flagged the *racR*-divergent *ydaST* genes of the Rac prophage as candidates, and the encoded polypeptides are still listed in PFAM (5) (PF15943, PF06254) as a toxin-antitoxin pair, despite a subsequent 2010 study finding no evidence for this (6). The two new RacR studies published in *mSphere* focus solely on the toxicity of these two genes at the expense of any insight into their toxicity, which could have been addressed by studying the corresponding loci in other temperate prophages of E. coli. Many prophages genes when ectopically expressed are detrimental to the bacterium but are likely to be beneficial (for the phage) during prophage induction. In the majority of lambdoid prophages, there are two functionally (but not always genetically) conserved transcriptional regulators divergently encoded from the phage repressor, the so-called phage immunity region (7). In the prototypical lambda phage, this locus encodes cI (repressor), Cro (lytic regulator), and cII (lysogenic regulator).

YdaS and YdaT were identified as Cro and cII homologs in the Rac prophage by Casjens (8) in 2003 and are also annotated as such in many databases, e.g., EcoGene (9). Coordinated expression of the lambda regulatory genes is essential for the lysis/lysogeny decision (10, 11), where the relative levels of Cro and cII proteins are controlled by both environmental inputs and other phage regulatory proteins (12–14). As recently shown for *ydaT* (15), overexpression of *cII* is highly toxic to E. coli (16, 17), yet Cro and cII are not considered toxins. It is highly likely that *racR*, *ydaS*, and *ydaT* form a similarly functioning regulatory locus. The *rac* regulatory circuit is fully functional—zygotic induction of prophage recombination genes occurs upon Hfr transfer of the *rac* locus to naive cells (18). That 1973 study identified the *rac* locus as the Rac prophage, named for recombination activation. Zygotic induction is lethal in up to 98% of recipient bacteria (19). Prophage repressors are essential only in the context of the lysogen (two other essential genes encode putative repressors of the defective prophages Qin and e14 [9]), and to focus on the phenotype of a particular locus without addressing its genetic context has led to what I consider the erroneous conclusion that the role of RacR is solely to repress the *ydaST* “toxin” genes—specifically, that the *ydaST-racR* module forms a “toxin-repressor” combination (1)—or that RacR-YdaS-YdaT form an atypical toxin-antitoxin system (2). Most likely, YdaS and YdaT proteins, while toxic to the host bacterium when overproduced or ectopically expressed, are actually essential regulatory components of the prophage genome.
